# Intravascular ultrasound–derived calcium score to predict stent underexpansion in moderately to severely calcified lesions

**DOI:** 10.1007/s12928-026-01327-6

**Published:** 2026-07-31

**Authors:** Masaomi Gohbara, Masahiko Noguchi, Yasuhiro Nakano, Yohei Sotomi, Kouji Yamamoto, Kyoko Hattori, Shun Kitajima, Yohei Hanajima, Katsuhiko Tsutsumi, Hidekuni Kirigaya, Jin Kirigaya, Shinnosuke Kikuchi, Hidefumi Nakahashi, Kensuke Matsushita, Kozo Okada, Noriaki Iwahashi, Masami Kosuge, Teruyasu Sugano, Toshiaki Ebina, Kiyoshi Hibi

**Affiliations:** 1 Division of Cardiology, Yokohama City University Medical Center, 4-57 Urafunecho, Minami-ku, Yokohama, 232-0024 Japan; 2 Department of Cardiology, Tokyo Bay Urayasu Ichikawa Medical Center, Urayasu, Japan; 3 Department of Cardiovascular Medicine, Kyushu University Hospital, Fukuoka, Japan; 4 Department of Cardiovascular Medicine, The University of Osaka Graduate School of Medicine, Osaka, Japan; 5 Department of Biostatistics, Yokohama City University, Yokohama, Japan; 6 Department of Laboratory Medicine and Clinical Investigation, Yokohama City University Medical Center, Yokohama, Japan; 7 Department of Cardiology, Yokohama City University Graduate School of Medicine, Yokohama, Japan

**Keywords:** Calcium score, Intravascular ultrasound, Optical coherence tomography, Percutaneous coronary intervention

## Abstract

The original intravascular ultrasound (IVUS) calcium score in percutaneous coronary intervention (PCI) applies only to lesions with a maximum calcium angle > 270°, distinguishing it from the optical coherence tomography (OCT) calcium score. We aimed to investigate a new IVUS-derived calcium score to predict stent underexpansion in moderately to severely calcified lesions. We retrospectively analyzed 238 IVUS-guided PCI cases with moderate to severe calcification visible on angiography, including 136 cases with IVUS-guided PCI as a derivation cohort and 102 cases with both IVUS- and OCT-guided PCI as a validation cohort, excluding cases in which atherectomy was used. In the derivation cohort, IVUS-derived maximum calcium angle showed significant area under the curve (AUC) to predict stent underexpansion (< 70%) at the maximum superficial calcium site (cutoff value: 228°, AUC: 0.72). A multivariable linear regression model showed that maximum calcium angle of 225° (regression coefficient: -11.69; *p* = 0.023) and the presence of calcified nodule (regression coefficient: -4.913; *p* = 0.004) were associated with stent expansion. A new IVUS calcium score (0–4 points) was defined as 3 points for maximum calcium angle > 225°, and 1 point for presence of a calcified nodule. A cutoff of ≥ 3 showed a good AUC (0.751) and an excellent negative predictive value (0.963) to predict stent underexpansion. In the validation cohort, the incidence of stent underexpansion was 0%, 0%, 8.6%, and 33.3% in patients with the new IVUS calcium score of 0, 1, 3, and 4, respectively (*p* = 0.004). The new IVUS-derived calcium score can effectively predict stent expansion in moderately to severely calcified lesions.

**Graphical Abstract:**

**Figure:**
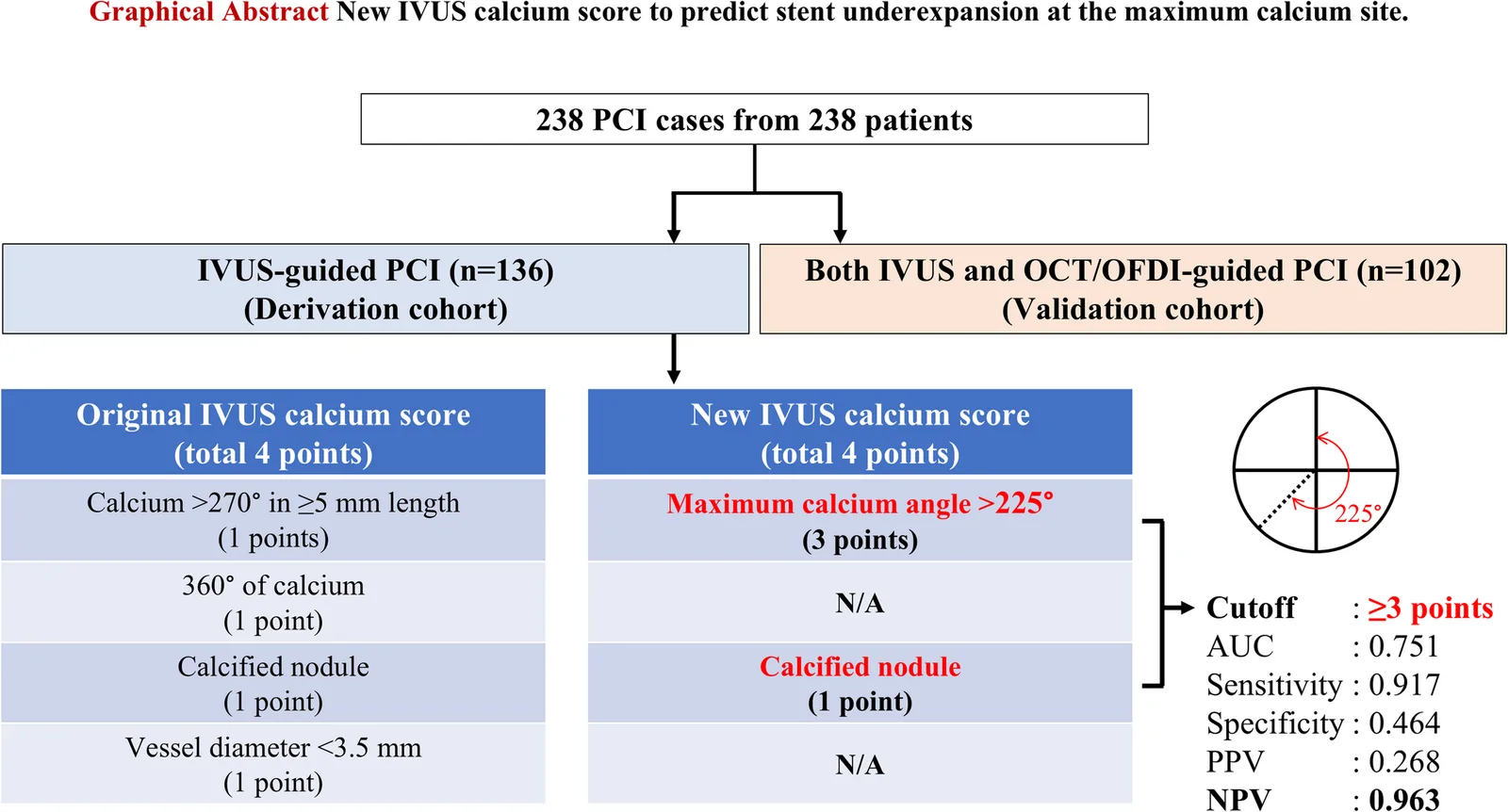

## Introduction

Coronary artery calcification (CAC) is a challenging subset of lesions in percutaneous coronary intervention (PCI) for patients with coronary artery disease [1, 2]. Occasionally a special balloon, an atherectomy device, or intravascular lithotripsy (IVL) is used to avoid stent underexpansion. In addition to the angiographic evidence of calcification, intravascular imaging is effective for predicting stent underexpansion [3–5]. Optical coherence tomography (OCT), an intravascular imaging modality, can assess the calcium angle, thickness, length, and morphology, and may have a theoretical advantage in predicting stent underexpansion [6, 7]. Intravascular ultrasound (IVUS) is another intravascular imaging modality capable of assessing calcium angle, length, and morphology, but not calcium thickness [8]. Recently, both OCT-based and IVUS-derived calcium scoring systems were developed to predict stent expansion during PCI when performed without using an atherectomy device [3, 4]. Although the two calcium scoring systems range from 0 to 4 points, significant differences exist between the methods used to develop the two models. The OCT-based calcium score targets all calcified lesions on OCT, while the IVUS-derived calcium score targets extremely severe calcified lesions with the maximum calcium angle > 270°. In addition, the OCT-based calcium score predicts stent underexpansion at the minimum stent area (MSA), whereas the IVUS-derived calcium score predicts stent underexpansion at the maximum superficial calcium site. Furthermore, each component of the two calcium scoring systems is different. Finally, an OCT-based calcium score of 4 and an IVUS-derived calcium score of ≥ 2 predict stent underexpansion. Thus, there is a discrepancy between OCT-based calcium scores and IVUS-derived calcium scores [9, 10]. 

Upon developing the original IVUS-derived calcium scoring system, it was limited to patients with extremely severe calcified lesions with maximum calcium angle > 270° on IVUS [4]. However, when a calcified lesion is visible on coronary angiography (CAG), intravascular imaging is performed to consider using atherectomy devices or IVL, irrespective of the maximum calcium angle > 270°. Therefore, a calcium scoring system should be developed for moderate or severe calcification visible on CAG, without limitation to cases with maximum calcium angle > 270°. In addition, a model that predicts stent expansion at the maximum superficial calcium site seems plausible to determine whether atherectomy devices or IVL are required. Furthermore, IVUS is more widely used than OCT because it does not require contrast, allows for easier evaluation of distal lesions even in severe stenotic lesions, and carries a lower risk of worsening dissection. Accordingly, IVUS was often used to guide atherectomy devices or IVL.

These considerations highlight the need to establish a new IVUS-derived calcium scoring system to predict stent expansion at the maximum superficial calcium site in moderately to severely calcified lesions. In this study, we aimed to investigate a new IVUS-derived calcium scoring system that could predict stent expansion in moderately to severely calcified lesions.

## Methods

### Study population

**Fig. 1:**
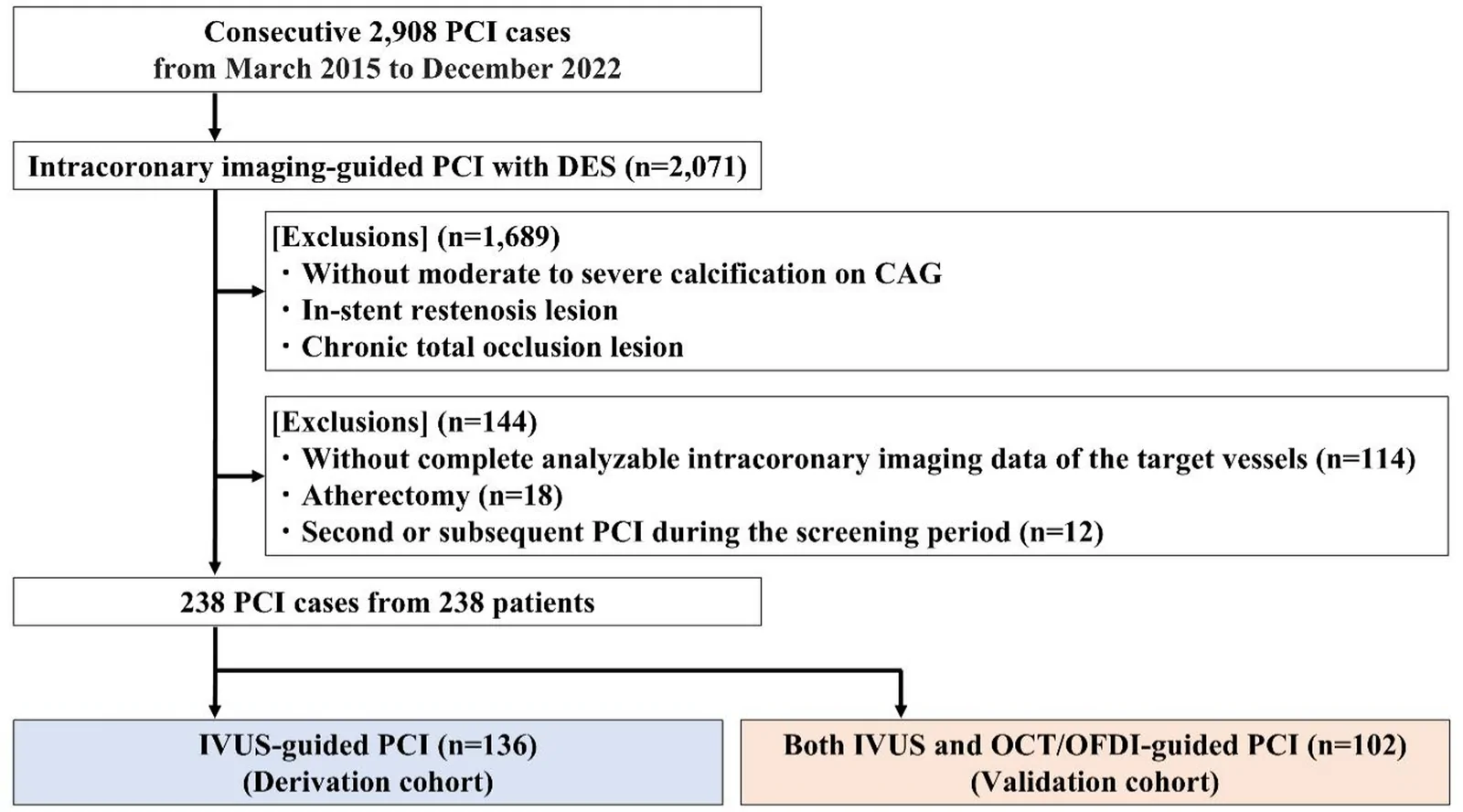
Study flowchart. Among 238 PCI cases, 136 cases with IVUS-guided PCI were included as a derivation cohort and 102 cases with both IVUS and OCT/OFDI-guided PCI were included as a validation cohort. IVUS, intravascular ultrasound; OCT, optical coherence tomography; OFDI, optical frequency domain imaging; PCI, percutaneous coronary intervention

This retrospective, observational study included patients with coronary artery disease who underwent PCI at Yokohama City University Medical Center. In Japan, intracoronary imaging is used in over 80% of all PCI cases [11], supported by strong evidence for improved clinical outcomes and full insurance coverage. In this study, we screened 2,908 PCI cases encountered between March 2015 and December 2022 in the era when IVL was not available in Japan (Fig. 1). The exclusion criteria were as follows: (1) without moderate to severe calcification on CAG; (2) in-stent restenosis lesion; (3) chronic total occlusion lesion; (4) without complete analyzable intracoronary imaging data of the target vessels; (5) previous atherectomy; and (6) second or subsequent PCI during the screening period. Among 2,908 PCI cases, we included 2,071 patients who received intracoronary imaging-guided drug-eluting stent (DES) implantation. Based on the exclusion criteria, 1,833 patients were excluded; 238 intracoronary imaging-guided PCI cases with DES and moderate or severe calcification visible on CAG in the target vessels of PCI, but not synonymous with angiographic evidence of calcification, were included in this study (Fig. 1). Among 238 PCI cases, 136 cases with IVUS-guided PCI were included as a derivation cohort, and 102 of 110 cases from our recent paper, excluding eight cases that underwent atherectomy, were included as a validation cohort [12]. 

We conducted the study in accordance with the Declaration of Helsinki, and it was approved by the Institutional Review Board of the Yokohama City University (approval number: F240100009). The requirement for individual informed consent was waived in accordance with the “opt-out” principle; however, patients could opt out of the database if they wished to do so.

### Angiographic evidence of calcification

In the United States, angiographic evidence of calcification was the indication criterion for IVL. It is defined by: (a) angiographic evidence of fluoroscopic radiopacities noted without cardiac motion prior to contrast injection, involving both sides of the arterial wall in at least one location and a total calcification length of at least 15 mm, extending partially into the target lesion, or (b) IVUS or OCT/optical frequency domain imaging (OFDI) imaging findings of calcium angle ≥ 270° on at least one cross-sectional image [13, 14]. 

### Intracoronary imaging

After intracoronary injection of 2–3 mg of isosorbide dinitrate, intracoronary imaging was performed using a standard technique with commercially available imaging systems with automated pullback. The IVUS images were acquired using standard techniques with OptiCross 40 MHz imaging catheter series (Boston Scientific, Marlborough, MA, USA), ViewIT 40 MHz imaging catheter series (TERUMO, Tokyo, Japan), or AltaView 40–60 MHz imaging catheter series (TERUMO, Tokyo, Japan) before PCI (pre-PCI) and after PCI (post-PCI). If an IVUS imaging catheter cannot pass through the target lesion, balloon dilatation with small balloons (1.5–2.0 mm) or a guide catheter extension was used.

### Intracoronary imaging analysis

Intracoronary imaging data were anonymized, and analysis was performed by three experienced investigators using echoPlaque 4.3 (INDEC Medical Systems, Inc, Mountain View, CA). The target lesion was defined as the stented segment. The original IVUS calcium score was evaluated using pre-PCI IVUS images based on: (1) a calcium angle > 270° over ≥ 5.0 mm length; (2) presence of superficial circumferential calcium (360°); (3) presence of a calcified nodule; and (4) small vessel size (< 3.5 mm) at the maximum superficial calcium site on a scale of 0 to 4, in accordance with a previous study [4]. 

In IVUS analysis, the presence of calcium was defined as bright echoes that obstructed the penetration of ultrasound, a phenomenon known as acoustic shadowing. Superficial calcium was defined as the leading edge of the acoustic shadow appearing within the shallowest 50% of the combined plaque and media thickness [8]. Using the pre-PCI IVUS images, the minimum lumen area, maximum superficial calcium angle, total continuous superficial calcium length, total length of continuous superficial calcium angle > 270°, total length of continuous superficial calcium angle > 180°, and vessel diameter at the maximum superficial calcium site were measured. The vessel diameter was measured at the site closest to the maximum superficial calcium site because the vessel diameter at the maximum superficial calcium site was difficult to measure owing to the acoustic shadow. Using the post-PCI IVUS images, the MSA and stent area at the maximum superficial calcium site were measured. The proximal and distal references were defined as the largest lumen and least plaque burden within 5 mm from the stent edge with no major intervening branches. Stent expansion was defined as the stent area divided by the average of the proximal and distal reference lumen areas and was calculated at both the MSA and maximum superficial calcium site. Stent underexpansion was defined as a stent expansion of < 70% [4]. 

### Statistical analysis

Continuous variables are expressed as mean ± standard deviation for normally distributed variables and as median (interquartile range) for skewed variables. Differences between the two groups were compared using the Student’s t-test for normally distributed variables, Wilcoxon rank-sum test for skewed variables, and chi-squared test or Fisher’s exact test, as appropriate, for categorical variables. Intra- and inter-observer variability of the image analysis was assessed by evaluation of 30 randomly selected images for the maximum calcium angle, calcium length, and MSA. Comparisons were performed by the same cardiologist at 6 months after the initial evaluation, as well as by two independent investigators. Intra- and inter-observer reproducibility of image analyses was assessed by intraclass correlation coefficient (ICC). Receiver operating characteristic (ROC) analyses showed the area under the curve (AUC) as the C statistic, with AUC = 0.50 (no accuracy) and AUC = 1.00 (maximal accuracy). The optimal cutoff values of the ROC analyses were determined using the Youden index (sensitivity+specificity − 1) without adjusting for covariates. A multivariable linear regression model with previously reported predictors was utilized to determine predictors of stent expansion (continuous variable) at the maximum superficial calcium site in the derivation cohort [3, 4]. To simplify the scoring system and harmonize the scale of regression coefficients, continuous variables were divided by clinically useful numerical values close to the cutoff value if they significantly predicted stent underexpansion, as well as the original OCT calcium scoring system [3]. Nominal variables were converted to dummy numerical values and used to determine the respective regression coefficients in the multivariate linear regression model of stent expansion. Next, a new IVUS calcium scoring system was developed based on the regression coefficients. Calibration plots were used to compare the predicted stent expansion derived from the multivariable linear regression model and the observed stent expansion to assess the prediction distribution of the model. After dividing patients from the two cohorts (total cohort) into quartiles of IVUS-derived maximum calcium angle (0–90°, 91–180°, 181–270°, and 271–360°), a significant overall difference was detected using the Fisher’s exact test. All statistical tests were two-tailed, and *p* < 0.05 was considered statistically significant. JMP^®^ Student Edition (SAS Institute Inc., Cary, NC, USA) was used for all the statistical analyses.

## Results

### Baseline characteristics

Among 136 PCI cases included in the derivation cohort, the median age was 78 (70–84) years, and 83 (61%) patients were male (Table 1). The proportion of patients with acute coronary syndrome, as well as hemoglobin levels and estimated glomerular filtration rates, were significantly lower in the derivation cohort than in the validation cohort (Table 1). The proportion of angiographic evidence of calcification, the original IVUS calcium score, maximum calcium angle, and calcium length were not significantly different between the two cohorts (Table 2). Stent underexpansion at the maximum superficial calcium site was observed more frequently in the derivation cohort than in the validation cohort (18% versus 7%, *p* = 0.014) (Table 2).

**Table 1:** Baseline characteristics

Variables	Derivation cohort	Validation cohort	*p* Value
	(*n* = 136)	(*n* = 102)	
Age (years)	78 (70–84)	75 (69–81)	0.084
Male sex	83 (61)	74 (73)	0.063
Body mass index (kg/m^2^)	22.9 (20.8–24.8)	23.0 (21.5–25.1)	0.393
Hypertension	114 (84)	85 (83)	0.920
Diabetes mellitus	67 (49)	45 (44)	0.431
Dyslipidemia	111 (82)	80 (78)	0.541
Prior MI	30 (22)	9 (9)	0.006
ACS	57 (42)	59 (58)	0.015
Target vessel			< 0.001
Right coronary artery	32 (24)	11 (11)	
Left main trunk	7 (5)	3 (3)	
Left anterior descending coronary artery	76 (56)	84 (82)	
Left circumflex coronary artery	21 (15)	4 (4)	
Graft	0 (0)	0 (0)	
Total number of stents in this PCI	1 (1–2)	1 (1–2)	0.748
Total length of stents in this PCI (mm)	29 (22–43)	33 (28–48)	0.042
Minimum stent diameter in this PCI (mm)	3.00 (2.50–3.00)	3.00 (2.75–3.50)	0.121
Hemoglobin (g/dL)	11.4 ± 1.8	12.2 ± 1.5	< 0.001
eGFR (mL/min/1.73m^2^)	47.3 (33.4–60.0)	61.0 (50.6–71.6)	< 0.001

Categorical variables are presented as number (percentage), and continuous variables are presented as mean ± standard deviation (SD) for normally distributed variables and as median (interquartile range [IQR]) for skewed variables

ACS, acute coronary syndrome; eGFR, estimated glomerular filtration rate; MI, myocardial infarction; PCI, percutaneous coronary intervention

**Table 2:** Calcification analysis and IVUS data analysis

Variables	Derivation cohort (*n* = 136)	Validation cohort (*n* = 102)	*p* Value
Angiographic evidence of calcification	86 (63)	68 (67)	0.584
IVUS calcium score	1 (0–2)	0 (0–1)	0.758
Calcium angle > 270° in ≥ 5.0 mm length	12 (9)	19 (19)	0.026
Presence of superficial circumferential (angle = 360°) calcium	23 (17)	28 (27)	0.050
Presence of a calcified nodule	23 (17)	9 (9)	0.070
Small vessel size (< 3.5 mm) at the maximum superficial calcium site	42 (31)	19 (19)	0.032
Minimum lumen area (mm^2^)	1.83 (1.52–2.24)	1.89 (1.49–2.22)	0.802
Maximum calcium angle (°)	253 (192–319)	274 (190–360)	0.383
Calcium length (mm)	14.0 (9.0–20.0)	14.3 (10.0–20.9)	0.456
Calcium length of calcium angle > 180°	2.0 (0.1–4.6)	2.9 (0.3–6.5)	0.077
Calcium length of calcium angle > 270°	0 (0–2.0)	0.8 (0–3.5)	0.025
Vessel size at the maximum superficial calcium site (mm)	3.83 ± 0.62	3.98 ± 0.61	0.054
Minimum stent area (mm^2^)	6.07 (4.69–7.05)	6.06 (4.83–7.30)	0.999
Stent area at the maximum superficial calcium site (mm^2^)	6.68 (5.58–8.09)	6.93 (6.15–8.55)	0.165
Average reference lumen area (mm^2^)	7.85 (6.67–9.44)	8.20 (7.15–9.63)	0.128
Stent expansion at the maximum superficial calcium site (%)	86.7 ± 14.9	85.8 ± 11.7	0.993
Stent expansion at the minimum stent area (%)	75.2 ± 11.7	71.9 ± 10.8	0.020
Stent underexpansion at the maximum superficial calcium site	24 (18)	7 (7)	0.014
Stent underexpansion at the minimum stent area	51 (38)	48 (47)	0.139

Categorical variables are presented as number (percentage), and continuous variables are presented as mean ± standard deviation (SD) for normally distributed variables and as median (interquartile range [IQR]) for skewed variables

IVUS, intravascular ultrasound

### Data reproducibility

The intra- and inter-observer variability in the image analysis of maximum calcium angle, calcium length, and MSA was assessed using ICC. The intra- and inter-observer variability for maximum calcium angle was 0.99 and 0.96, for calcium length was 0.99 and 0.94, and for MSA was 0.97 and 0.93, respectively.

### New IVUS calcium score

**Fig. 2:**
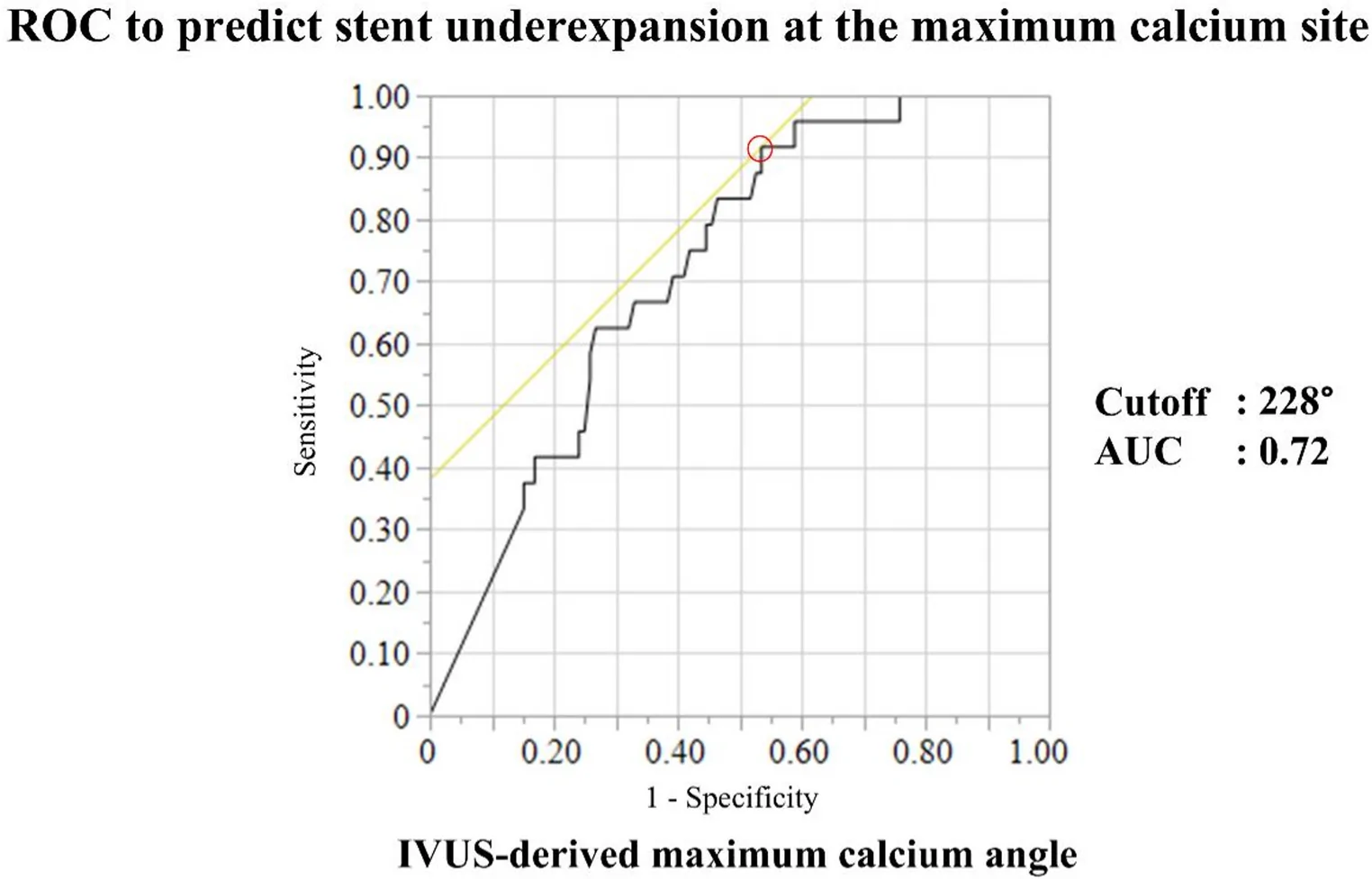
ROC analysis to predict stent underexpansion at the maximum superficial calcium site in the derivation cohort. IVUS-derived maximum calcium angle showed significant AUC to predict stent underexpansion (< 70%) at the maximum superficial calcium site (cutoff value: 228°, AUC: 0.72) AUC, area under the curve; IVUS, intravascular ultrasound; ROC, receiver operating characteristic

**Fig. 3:**
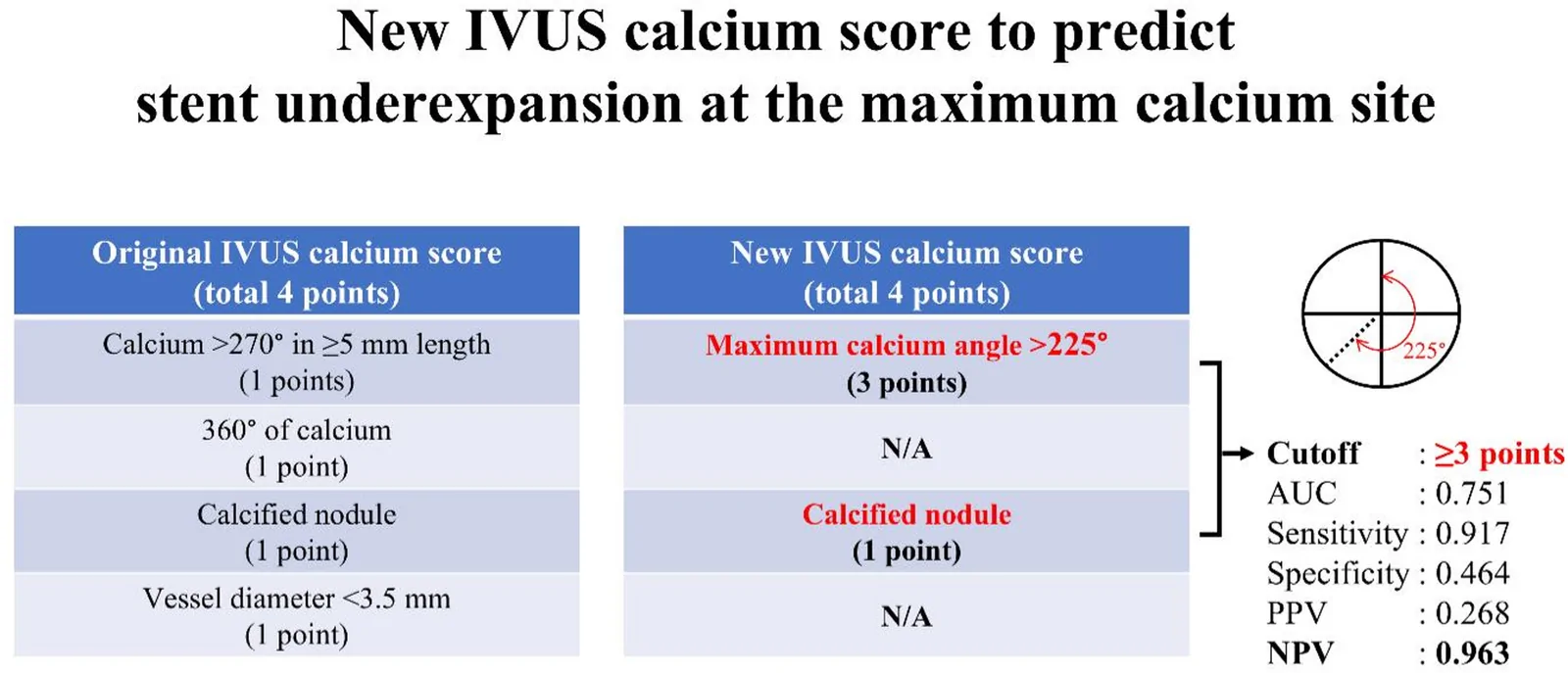
New IVUS calcium score. A new IVUS calcium score was developed from the derivation cohort consisting of the IVUS-derived maximum calcium angle > 225° and calcified nodule. The IVUS-derived maximum calcium angle > 225° was allocated a score of 3 and calcified nodule was allocated a score of 1, resulting in a new IVUS calcium score of 4. A cutoff of ≥ 3 demonstrated a good AUC (0.751) and an excellent negative predictive value (0.963) to predict stent underexpansion. AUC, area under the curve; IVUS, intravascular ultrasound

**Fig. 4:**
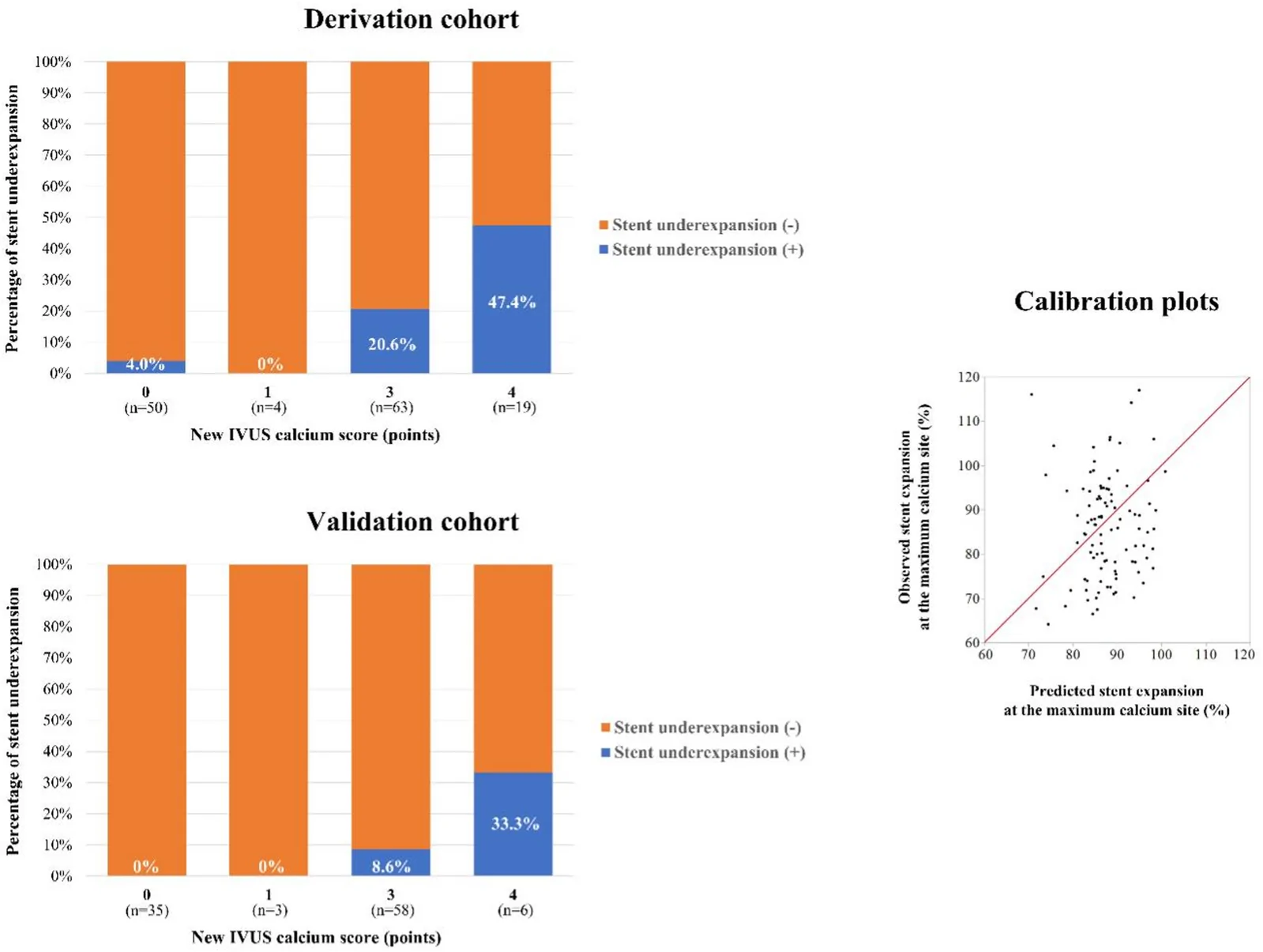
Incidence of stent underexpansion by the new IVUS calcium score in the derivation and validation cohorts. In the derivation cohort, the incidence of stent underexpansion at the maximum calcium site was 20.6% in patients with the new IVUS calcium score of 3, and 47.4% in those with the new score of 4. In the validation cohort, the incidence of stent underexpansion at the maximum calcium site was 8.6% in patients with the new IVUS calcium score of 3, and 33.3% in those with the new score of 4. Calibration plots showed reasonable correlation between the predicted stent expansion and observed stent expansion. IVUS, intravascular ultrasound

In the derivation cohort, IVUS-derived maximum calcium angle showed a significant AUC to predict stent underexpansion at the maximum superficial calcium site (cutoff value: 228°, AUC: 0.72) (Fig. 2). Consequently, 225°, which is close to the maximum calcium angle cutoff value of 228°, was used in the multivariable linear regression model. A multivariable linear regression model showed that the predictors of stent expansion were maximum calcium angle per 225° (regression coefficient: -11.69; *p* = 0.023) and presence of a calcified nodule (regression coefficient: -4.913; *p* = 0.004) (Table 3). Based on these regression coefficients, the IVUS-derived maximum calcium angle > 225° was allocated a score of 3, and the calcified nodule was allocated a score of 1, resulting in a new IVUS calcium score of 4 (Fig. 3). A cutoff of ≥ 3 showed a good AUC of 0.751 and an excellent negative predictive value of 0.963 to predict stent underexpansion. In the derivation cohort, the incidence of stent underexpansion at the maximum calcium site was 20.6% in patients with the new IVUS calcium score of 3, and 47.4% in those with the new score of 4 (Fig. 4). In the validation cohort, the incidence of stent underexpansion at the maximum calcium site was 8.6% in patients with the new IVUS calcium score of 3, and 33.3% in those with the new score of 4 (Fig. 4). Calibration plots showed reasonable correlation between the predicted stent expansion and observed stent expansion (Fig. 4).

**Table 3:** Predictors of stent expansion (continuous variable) at the maximum superficial calcium site using a multivariable linear regression model in the derivation cohort

Variables	Regression coefficient (95% CI)	*p* Value
Maximum calcium angle, per 225°	-11.690 (-21.717 – -1.663)	0.023
Calcium length, per 1 mm	-0.105 (-0.419–0.209)	0.510
Vessel size at the maximum superficial calcium site, per 1 mm	3.169 (-0.811–7.149)	0.118
Presence of superficial circumferential (angle = 360°) calcification	0.564 (-3.575–4.703)	0.788
Presence of a calcified nodule	-4.913 (-8.176 – -1.649)	0.004

CI, confidence interval

### Incidence of stent underexpansion at the maximum calcium site using the cutoff of IVUS calcium score

**Fig. 5:**
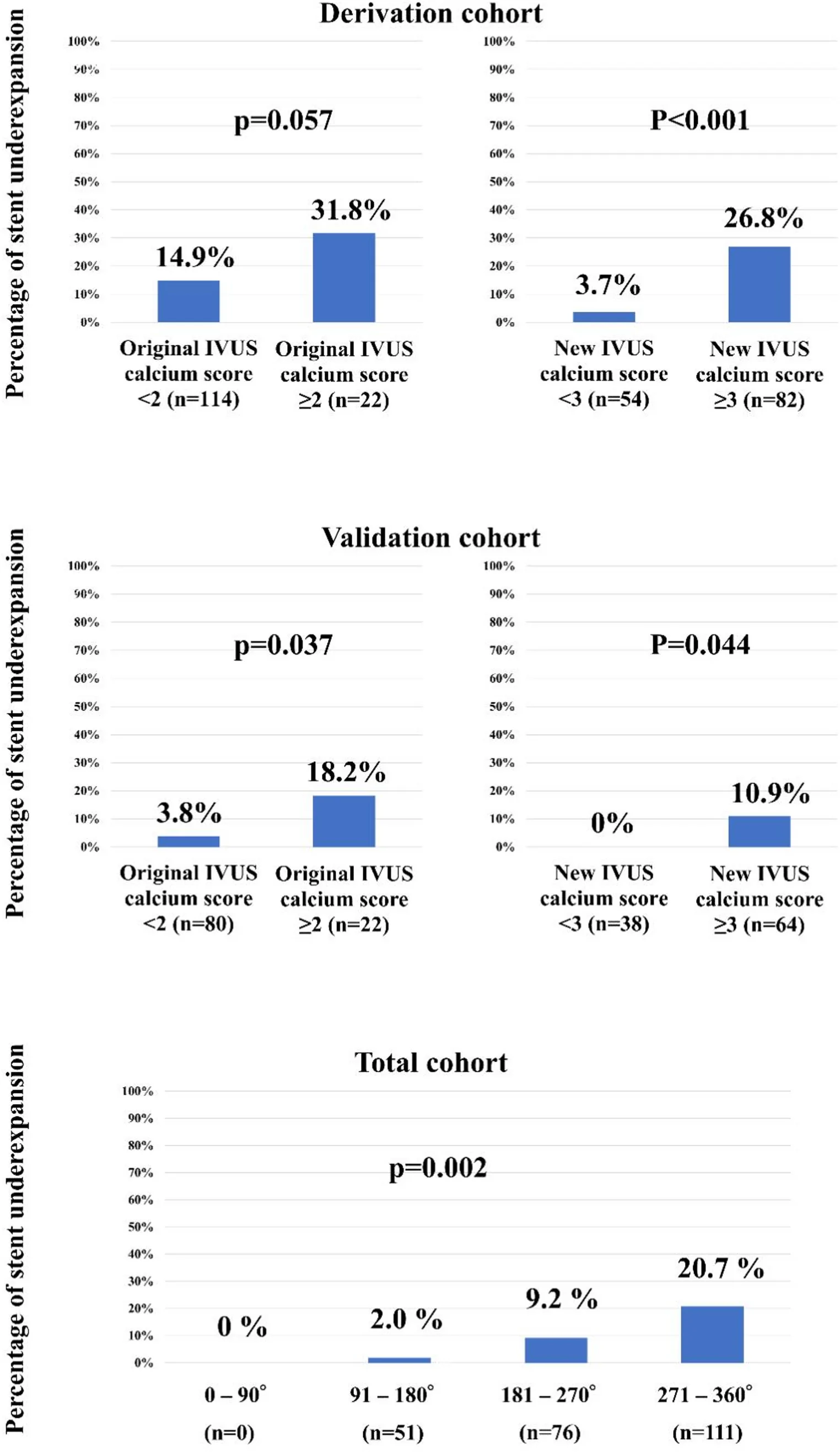
Incidence of stent underexpansion at the maximum calcium site. In the derivation cohort, patients with the original IVUS calcium score ≥ 2 did not have a statistically higher incidence of stent underexpansion at the maximum calcium site than those with the original score < 2. However, patients with the new IVUS calcium score ≥ 3 had a higher incidence of stent underexpansion than did those with the new score < 3 (26.8% versus 3.7%, *p* < 0.001). In the validation cohort, patients with the original IVUS calcium score ≥ 2 had a higher incidence of stent underexpansion than did those with the original score < 2 (18.2% versus 3.7%, *p* = 0.037). Similarly, patients with the new IVUS calcium score ≥ 3 had a higher incidence of stent underexpansion than did those with the new score < 3 (10.9% versus 0%, *p* = 0.044). In the total cohort, there were significant differences in the incidence of stent underexpansion among the quartiles (*p* = 0.002). IVUS, intravascular ultrasound

In the derivation cohort, patients with the original IVUS calcium score ≥ 2 did not have a statistically higher incidence of stent underexpansion at the maximum calcium site than those with the original score < 2 (*p* = 0.057), which likely due to the small sample size of the patients with original calcium score ≥ 2 (*n* = 22). However, patients with the new IVUS calcium score ≥ 3 had a higher incidence of stent underexpansion than did those with the new score < 3 (26.8% versus 3.7%, *p* < 0.001) (Fig. 5).

In the validation cohort, patients with the original IVUS calcium score ≥ 2 had a higher incidence of stent underexpansion than did those with the original score < 2 (18.2% versus 3.7%, *p* = 0.037). Similarly, patients with the new IVUS calcium score ≥ 3 had a higher incidence of stent underexpansion than did those with the new score < 3 (10.9% versus 0%, *p* = 0.044) (Fig. 5).

### Incidence of stent underexpansion at the maximum calcium site using quartiles of IVUS-derived maximum calcium angle in the total cohort

Subsequently, we compared the incidence of stent underexpansion according to the quartiles of IVUS-derived maximum calcium angle in the total cohort (derivation and validation cohorts). Overall, there were significant differences in the incidence of stent underexpansion between quartiles (*p* = 0.002) (Fig. 5).

### Clinical outcomes

Finally, we compared clinical outcomes between the groups stratified based on the new IVUS calcium score (i.e. patients with the new IVUS calcium score < 3 versus those with calcium score ≥ 3). No difference in the incidence of clinical outcomes was observed between the groups across the total cohort of 238 patients (Table 4).

**Table 4:** Clinical outcomes according to the new IVUS calcium score in the total cohort

Clinical outcomes	New IVUS calcium score < 3 (*n* = 92)	New IVUS calcium score ≥ 3 (*n* = 146)	*p* Value
All cause death	6 (6.5)	19 (13.0)	0.112
Cardiac death	1 (1.1)	5 (3.4)	0.410
Myocardial infarction	5 (5.4)	7 (4.8)	0.826
Target vessel revascularization	1 (1.1)	4 (2.7)	0.651
Heart failure	3 (3.3)	8 (5.5)	0.537

Categorical variables are presented as number (percentage)

IVUS, intravascular ultrasound

## Discussion

In this study, we developed a new IVUS calcium score defined as 3 points for maximum calcium angle > 225° and 1 point for presence of a calcified nodule, with a total score of 0–4 points. A cutoff of ≥ 3 points showed a good AUC and an excellent negative predictive value to predict stent underexpansion. The new IVUS-derived calcium scoring system was developed to predict stent expansion in moderately to severely calcified lesions; therefore, it could be used widely in daily practice.

In Japan, the original IVUS calcium score or OCT calcium score of ≥ 3 is considered suitable for IVL [15]. However, the original IVUS calcium score was developed from cases limited to extremely severely calcified lesions. Based on the current indications for IVL with the original IVUS calcium score ≥ 3, IVL cannot be used even in patients in whom safe calcification treatment is required [10]. To fill this gap, we recently investigated a new IVUS-based criterion equivalent to OCT calcium score, and reported that an IVUS-derived maximum calcium angle > 225° or combined with IVUS-derived calcium angle > 180° in > 3 mm length could provide a useful alternative to OCT calcium score of ≥ 3 or 4 [12]. However, an IVUS-derived maximum calcium angle > 225° was determined as a surrogate marker to predict OCT calcium score of ≥ 3, but cannot predict stent expansion.(9) Therefore, in this study, we developed a new IVUS-derived calcium score to predict stent expansion in moderately to severely calcified lesions. Similar to our previous report [12], the IVUS-derived maximum calcium angle > 225°, which was reported to be an alternative to OCT-based calcium score of ≥ 3, was also a strong predictor of stent expansion. We allocated a score of 3 points if the calcium angle was over 225° in the new IVUS-derived calcium score. The results of this study may provide a rationale to use IVL during IVUS-guided PCI in cases in which IVL is considered desirable, or even in cases in which IVL was not indicated by the original IVUS-derived calcium score.

In Europe, an EAPCI clinical consensus statement in collaboration with the EURO4C-PCR group recommended that calcium modification with atherectomy devices or IVL is considered for lesions with an original IVUS calcium score ≥ 2 or OCT calcium score > 3 (i.e., 4) [16]. Similarly, in the United States, SCAI Expert Consensus Statement on the Management of Calcified Coronary Lesions recommended that calcium modification with atherectomy devices or IVL is considered for lesions with a 360° arc of calcium, > 270° arc of calcium over > 5 mm length, or additional high-risk imaging features including presence of a calcified nodule, lesion with external elastic lamina < 3.5 mm or negative remodeling, and minimum thickness of calcium > 0.5 mm on OCT [17]. These recommendations were based on OCT- and IVUS-derived calcium scoring systems; however, they were established based on different lesion backgrounds. The OCT-based calcium scoring system targeted cases in which calcification was detected by OCT and covers all calcified lesions, while the original IVUS-based calcium scoring system targeted only cases in which calcium angle > 270° was observed. Recently, a revised OCT-derived calcium scoring system, which was also limited to patients with extremely severe calcified lesions with the maximum calcium angle > 270° on OCT, was developed [18]. However, challenging cases that had poor stent expansion without atherectomy, even in lesions that were < 270°, were occasionally encountered [19, 20]. The randomized ROLLER COASTR-EPIC22 trial, which compared rotational atherectomy (RA), IVL, and excimer laser coronary angioplasty for the treatment of patients with calcified coronary lesions, included moderate to severe calcification visible on CAG without relying on a maximum calcium angle > 270° [21]. Additionally, a retrospective study comparing RA, orbital atherectomy (OA), and IVL for the treatment of patients with calcified coronary lesions also included severely calcified plaques with a maximum calcium angle of ≥ 180° but not > 270° on OCT. The mean maximum calcium angle was 234° for RA, 223° for OA, and 327° for IVL [22]. Furthermore, another retrospective study comparing DES with drug-coated balloon (DCB) after OA, the median maximum calcium angle was 222° in the DES group and 265° in DCB group [23]. Recently, several retrospective studies have reported that maximum calcium thickness, large and thick eccentric calcium, and negative remodeling were associated with stent underexpansion, even in non-severely calcified lesions with a maximum calcium angle < 180° [19, 20]. This reflects the actual clinical practice and demonstrates the need to consider atherectomy even in cases of maximum calcium angle ≤ 270°. Therefore, we believe that a calcium scoring system that targets calcified lesions not limited to > 270° is necessary.

Although a maximum calcium angle of ≥ 270° was conventionally used as one of the criteria for using atherectomy devices or IVL [13, 14], this angle was not directly derived from a model predicting stent underexpansion. The hallmark paper by Mintz et al. revealed that in most target lesions with a maximum calcium angle of ≥ 270°, calcium was detected by angiography, which may have established the threshold of 270° [24]. In the original paper on the IVUS calcium score, the authors expressed an opinion that a maximum calcium angle ≤ 180° did not affect stent expansion [4]. In the new OCT calcium score, the same research group changed the inclusion criteria previously used from all calcified lesions to extremely severely calcified lesions with a maximum calcium angle > 270° [18]. Here, we demonstrated a relatively high incidence of stent underexpansion in the 181–270° quartile of maximum calcium angle (9.2%). Therefore, we believe that > 270° is not an absolute criterion and propose a maximum calcium angle of more than 225° as a criterion for predicting stent underexpansion in moderately to severely calcified lesions.

The finding that a calcified nodule is a predictor of stent underexpansion in the original IVUS calcium score model is consistent with our results, where the new IVUS-derived calcium score of 4, defined as a combined maximum calcium angle > 225° and a calcified nodule, was most frequently associated with stent underexpansion. Generally, a superficial calcification sheet exists around a calcified nodule, and its maximum calcium angle is often large. However, in rare cases, a calcified nodule without a large maximum calcium angle also exists. In this study, cases with a calcified nodule and a maximum calcium angle of ≤ 225° were observed in 7 out of 238 cases (2.9%), but none of them resulted in stent underexpansion. This appears to contradict the original IVUS calcium score model, which indicated that a calcified nodule is a predictor of stent underexpansion; however, these cases were outside the range of the original IVUS calcium score model because their maximum calcium angle was ≤ 270°. When the maximum calcium angle is not large, even if a calcified nodule is present, stent expansion may be achieved in other areas, preventing stent underexpansion.

In the present study, we developed a new calcium scoring system, not limited to a maximum calcium angle > 270°, that can be used as a criterion to identify the need for calcium modification by atherectomy devices or IVL in PCI cases.

### Study limitations

This study had some limitations. First, this was a single-center, retrospective, observational study with a relatively small sample size. Second, we excluded cases of severe stenosis with severely calcified lesions in which the pre-PCI intracoronary imaging catheter could not cross. Third, we did not have an external cohort to confirm the validity of the new IVUS-derived calcium score. Fourth, data analyses of this study should be performed at an independent core-laboratory. We have started a multi-institutional prospective observational study to confirm our findings. As a sub-study of that study, we plan to measure the new IVUS calcium score at an independent core-laboratory and verify it as external validation. Fifth, this study did not demonstrate significant differences between the groups based on the new IVUS calcium score regarding clinical outcomes. This was likely attributable to the limited number of cases and events. One reason for the low incidence of events was our proactive use of optimal IVUS-guided pre- and post-dilatation to avoid stent underexpansion, even in cases of severe calcification.

## Conclusions

A new IVUS-derived calcium score can effectively predict stent expansion in moderately to severely calcified lesions.

## Declarations

### Data and/or Code availability

The deidentified participant data will not be shared.
